# Simultaneous multi-crop land suitability prediction from remote sensing data using semi-supervised learning

**DOI:** 10.1038/s41598-023-33840-6

**Published:** 2023-04-26

**Authors:** Amanjot Bhullar, Khurram Nadeem, R. Ayesha Ali

**Affiliations:** Department of Mathematics and Statistics, University of Guelph, Guelph, N1G 2W1 Canada

**Keywords:** Climate sciences, Environmental sciences, Mathematics and computing

## Abstract

Land suitability models for Canada are currently based on single-crop inventories and expert opinion. We present a data-driven multi-layer perceptron that simultaneously predicts the land suitability of several crops in Canada, including barley, peas, spring wheat, canola, oats, and soy. Available crop yields from 2013–2020 are downscaled to the farm level by masking the district level crop yield data to focus only on areas where crops are cultivated and leveraging soil-climate-landscape variables obtained from Google Earth Engine for crop yield prediction. This new semi-supervised learning approach can accommodate data from different spatial resolutions and enables training with unlabelled data. The incorporation of a crop indicator function further allows for the training of a multi-crop model that can capture the interdependences and correlations between various crops, thereby leading to more accurate predictions. Through k-fold cross-validation, we show that compared to the single crop models, our multi-crop model could produce up to a 2.82 fold reduction in mean absolute error for any particular crop. We found that barley, oats, and mixed grains were more tolerant to soil-climate-landscape variations and could be grown in many regions of Canada, while non-grain crops were more sensitive to environmental factors. Predicted crop suitability was associated with a region’s growing season length, which supports climate change projections that regions of northern Canada will become more suitable for agricultural use. The proposed multi-crop model could facilitate assessment of the suitability of northern lands for crop cultivation and be incorporated into cost-benefit analyses.

## Introduction

Agricultural land suitability prediction is the process of predicting the suitability of land in a given location to grow different types of crops with the objective of anticipating an area’s crop production potential and limitations^1^. Land suitability prediction is particularly important to Canada because it is suspected that Canada will become more suitable for agriculture as climate change progresses^2^. KC et al.^2^ estimated that 1.85 million km$^2$ of land will become suitable for agriculture by 2080 using models that relied on expert opinions. However, their qualitative analysis could not quantify the extent to which the land will be suitable.

It is challenging to distinguish lands that are similar in suitability with expert opinion-based models, such as multi-criteria decision models and multi-criteria evaluation, as they rely on the subjective judgment of experts rather than objective data^3–5^. Experts may have different opinions on what environmental variables are important in determining land suitability and may weigh these variables differently because of personal bias^6^. Accordingly, there is a limit on the level of precision that can be achieved with an expert opinion-based model. Lands with similar suitability scores are typically grouped into five classes ranging from “not suitable” to “highly suitable”, where the class definitions arise from different classification methods (e.g., equal interval ArcView classification, FAO definitions, etc.)^7–9^. Higher precision models may permit land suitability to be quantified over a continuous scale and, given the significant amount of agronomic geospatial data that is publicly accessible (e.g., via Google Earth Engine) it is worth investigating data-driven models^10^. A data-driven land suitability model along with climate projections can allow for future land suitability to be quantitatively predicted, thereby facilitating a more accurate comparison between the long term economic benefits and environmental downfalls of cultivating new lands^11^.

When crop yield data is not available at a sufficient level of detail, or there is altogether an insufficient amount of data, expert opinion-based methods may be preferable to data-driven ones^12^. Indeed, Radočaj et al.^12^ found that of the 186 studies on land suitability modelling published during the period of 2010–2020, 166 developed expert opinion-based models^2,12–14^. Ozkan et al.^13^ identified suitable areas for agriculture in the Central Anatolia Region by estimating land suitability as a weighted sum of select soil-landscape variables. Three experts were asked to judge the relative importance of the variables by making pairwise comparisons between them. A Fuzzy Analytic Hierarchy Process (FAHP) was applied to combine these pairwise comparisons and derive weights that estimated the relative importance of each variable for land suitability. These weights were then used in a weighted sum to compute suitability scores that mapped agricultural areas to suitability classes. Current practice in land suitability modelling still largely relies on expert opinions, with most models developed using the Analytic Hierarchy Process (AHP)^15–21^. However, incorporating crop yields or related indicators into land suitability models can help overcome the subjectivity and lack of model evaluation inherent in expert-based models.

Chemura et al.^11^ developed a model that maps soil and climate variables to a land suitability value derived from crop yield data. From our knowledge, this is the first work that used a data-driven method to model land suitability. Suitability classes were defined using the 25th, 50th, and 75th percentiles of the yields for maize, sorghum, cassava and groundnut in Ghana. Soil and climate variables were represented by summary statistics at the district level (e.g., mean, minimum, maximum) before each district is mapped to a suitability class. More recently, Ganesan et al. (2021)^22^ and Ismaili et al.^23^ compare several machine learning methods for crop-land suitability, including decision trees, random forests, neural networks, and support vector machines. Ganesan et al. (2021) used government data on soil and environmental characteristics for one district in Tamil Nadu, India (area: 2916 km$^2$) while Ismaili et al.^23^ combined soil samples with Google Earth Engine climate-landscape data for the Beni-Mellal-Khenifra region of central Morocco (area: 1541 km$^2$).

Unfortunately, using district level summary statistics can be problematic if, for example, the reporting district size resolution is low or reporting district sizes are not uniform. In Canada, the average yield of a crop is recorded over districts that are very large in area but for which only a small fraction of land may be used for farming, It is difficult to model the relationship between the district level soil-climate-landscape variables and crop yield for where the crop is actually grown within the district^24^. Further, each crop is typically modelled using separate single-crop models. Indeed, an inherent problem with land suitability prediction is that one must use features of the soil-climate-landscape data for where a given crop is cultivated to make inferences about the suitability on lands where the crop is not cultivated. Single-crop models, whether expert-opinion or machine learning based, suffer from this dilemma. However, for any given crop, there may be other crops that can grow under similar environmental conditions and a more robust approach may be to use a multi-crop model that shares information across crops.

A multi-crop model simultaneously predicts yield for multiple crops and can exploit soil-climate-landscape data for crops that can grow in the same regions. Such a model could potentially improve accuracy because it uses a larger training set whilst retaining a similar number of model parameters. By sharing information across crops that can grow under comparable conditions, the model would be forced to learn a set of parameters that work well for predicting the yields of many crops which, in a way, can create a regularizing effect and mitigate overfitting. Although multi-crop models have not been used in land suitability prediction, they have been used for predicting crop yield at harvest using early stage crop growth, with an objective to help farmers make real-time decisions that maximize yield^25^. Khaki et al.^25^ developed a multi-crop yield prediction model for corn and soy based on deep transfer learning. Observed yields for each crop at the district (county) level over several years was available as input, along with discretized satellite images and crop-specific land cover data. Their model is promising, particularly for crops that are grown in similar regions, as is the case for corn and soy. However, the data demand for developing a multi-crop model is high with conventional training methods, as in Khaki et al.^25^. Hundreds of districts were used from any one year, districts were small and rather homogeneous compared to that available for Canada, and the yields of soy and corn were observed in each district.

It is challenging to model land suitability with district-level mean crop yields when annual crop inventory maps are unavailable. The soil-climate-landscape variables of the plots of land that contribute to the mean recorded yields would be unknown, in turn making it difficult to model the relationship between soil-climate-landscape variables and land suitability. Alternatively, Ismaili et al.^23^ and Mostafiz et al.^26^ use vegetation indices that are available at the farm level as a proxy for crop yield. Vegetation indices provide information about the greenness of a plot of land, which typically reaches its peak during a particular stage of crop growth. This peak level of greenness is generally indicative of the yield that will be obtained during harvest^23,26^. However, vegetation indices may not be comparable across different plots of land due to differing background levels of greenness caused by other factors such as canopy, soil, or atmospheric conditions. Moreover, a severe weather occurrence, or the presence of pests or disease can disrupt the connection between the vegetation index and the crop yield at harvest^27,28^. As a result, it can be challenging for the model to accurately learn the relationship between soil-climate-landscape variables and land suitability. One may prefer to use observed crop yield data when it is available.

The primary objective of the current work is to develop a fully data driven multi-crop land suitability model for Canada, based on neural networks, by using crop yield as a proxy for suitability. Our model takes agronomic variables as input (per Table 1) and outputs continuous land suitability scores for each of several crops simultaneously. Another challenge in developing a multivariate model is that the yield of all crops must be observed for a given set of soil-climate-landscape variables but only a single type of crop is grown at any location during the growing season. We overcome these problems by implementing semi-supervised learning and using an indicator function for the crop grown at a given place/time in calculating the neural network loss. Consequently, the yields of different crop types do not have to be observed for every set of input variables.

We present a new method of using soil-climate-landscape variables at the farm level to downscale observed average yields from the district level to the farm level, thereby directly modelling land suitability with recorded yields. Our model is capable of handling data with varying spatial resolutions and does not rely on reducing district-level soil-climate-landscape data into summary statistics over the spatial domain.

## Methods

### Crop yield dataset

The soil-climate-landscape variables used as input (see Table 1) were informed by previous land suitability models that tend to adequately discriminate which crops can be grown where and how well they can grow in those regions^11,13,14^. The geospatial images were obtained from Google Earth Engine^10^ and all soil variables, indexed 3–7 on Table 1, were obtained for soil depth 30 cm. The climate variables used for the fitting process were limited to mean precipitation rates, and maximum and minimum daily temperatures to ensure that the model is compatible with climate projections for future work. The climate variables were reduced to mean values calculated over particular periods of the growing season, see Table 1.

**Table 1:** Summary of soil-climate-landscape variables obtained from geospatial images from Google Earth Engine^10^

	**Short Name**	**Description**	**Data Source**	**Availability**	**Res.**
1	Slope	Slope	ALOS AW3D30	Fixed	30 m
2	Aspect	Aspect (orientation of slope)	ALOS AW3D30	Fixed	30 m
3	Soil water content	Soil water content	EnvirometriX Ltd	Fixed	250 m
4	Organic carbon	Soil organic carbon	EnvirometriX Ltd	Fixed	250 m
5	pH	Soil pH	EnvirometriX Ltd	Fixed	250 m
6	Bulk density	Soil bulk density	EnvirometriX Ltd	Fixed	250 m
7	Ttexture	Soil texture	EnvirometriX Ltd	Fixed	250 m
8	precipitation1	Mean of daily mean precipitation rate (March–April)	GPM	2000–2023	11132 m
9	precipitation2	Mean of daily mean precipitation rate (May–June)	GPM	2000–2023	11132 m
10	precipitation3	Mean of daily mean precipitation rate (July–Sept)	GPM	2000–2023	11132 m
11	max1	Mean of daily max air temperature (March–April)	GLDAS	2000–2023	27830 m
12	max2	Mean of daily max air temperature (May–June)	GLDAS	2000–2023	27830 m
13	max3	Mean of daily max air temperature (July–Sept)	GLDAS	2000–2023	27830 m
14	diurnal1	Mean of daily diurnal air temperature (March–April)	GLDAS	2000–2023	27830 m
15	diurnal2	Mean of daily diurnal air temperature (May–June)	GLDAS	2000–2023	27830 m
16	diurnal3	Mean of daily diurnal air temperature (July–Sept)	GLDAS	2000–2023	27830 m

Diurnal temperature is defined as the difference between the maximum and minimum daily temperature. The mean is calculated temporally, not spatially, for the time dependent variables. GPM and GLDAS are acronyms for Global Precipitation Measurement and Global Land Data Assimilation System, respectively. The GPM and GLDAS variables were available at a temporal resolution of every three hours. The Res. columns displays the spatial resolution of the images.

**Table 2:** An example of how the crop yield data is structured. Soil-climate-landscape variables are denoted by $x_1, x_2, \ldots , x_{16}$, are the inputs to the model while district yield is the label/response. The mean production of all observations (pixel IDs) that share the same crop, year, and district is assumed to equal the stated district yield as the yield is not known at the pixel level.

Pixel ID	Crop	Year	District Yield	District	$x_1$	$x_2$	$\ldots$	$x_{16}$
					(slope)	(aspect)	$\ldots$	(diurnal3)
1	Spring Wheat	2013	40 bushels/acre	Alberta 1	0.74	0.67	$\ldots$	0.88
2	Spring Wheat	2013		Alberta 1	0.54	0.72	$\ldots$	0.34
$$\vdots$$								
650	Spring Wheat	2013		Alberta 1	0.12	0.07	$\ldots$	0.97
651	Spring Wheat	2013	80 bushels/acre	Alberta 2	1.3	3.7	$\ldots$	0.7
652	Spring Wheat	2013		Alberta 2	0.47	0.07	$\ldots$	0.27
$$\vdots$$								
1134	Spring Wheat	2013		Alberta 2	0.17	0.34	$\ldots$	0.43
$$\vdots$$								

Statistics Canada offers data on the crop yields of several crops from 1976 to 2020, in units of bushels per acre. The yield data is available over large districts called Small Area Data regions (SADs), see Supplementary Fig. S2 online^29^. However, the crops are generally grown on a very small portion of any SAD. As a result, using the soil-climate-landscape features that cover an entire SAD as input to the model to predict crop yield will introduce too much noise. To combat this issue, the soil-climate-landscape features are masked to focus on the portions of a SAD where the crops are cultivated using the annual crop inventory data of Canada^30^. The annual crop inventory data is available for most of the provinces from 2013–2020. It is a 30 m resolution map that segments the land of Canada into land cover classes such as oats, barley, etc. and is at least 80% accurate for every province in a given year^30^. The map displays where particular crops were observed to grow. An example of how the crop yield data is structured for our modelling framework is given in Table 2, where the soil-climate-landscape variables are represented by *x*, with subscripts corresponding to the index given in Table 1.

Crop yield data from 2013 to 2020 will be used to train the model because the annual crop inventory maps are only available for that time frame. Unfortunately, Statistics Canada does not offer a geographic polygon file that identifies the SADs. Instead the SADs must be manually determined by matching different polygon files using a table provided by Statistics Canada. This was done twice; once for the time frame 2013–2016, and another for the time frame 2017–2020 because the SADs change every 5 years. To speed up learning, each soil-climate-landscape variable was standardized to have a mean of 0 and standard deviation of 1. The yield was also standardized in the same way. Standardizing data is an important step before training a machine learning model because it decreases the time required to train the model by ensuring that the weights are updated in a consistent and stable direction. If the input features are at different scales then the training algorithm may overshoot when updating the weights causing the model to oscillate to convergence, which will ultimately lead to slower training times^31^. Lastly, very large SADs were not used in the analysis due to their high computer memory requirements. These SADs include Alberta 60, Alberta 70, Ontario 5, Quebec 2, Quebec 10, and all SADs in British Columbia.

### Semi-supervised learning with a multivariate multilayer perceptron

**Figure 1:**
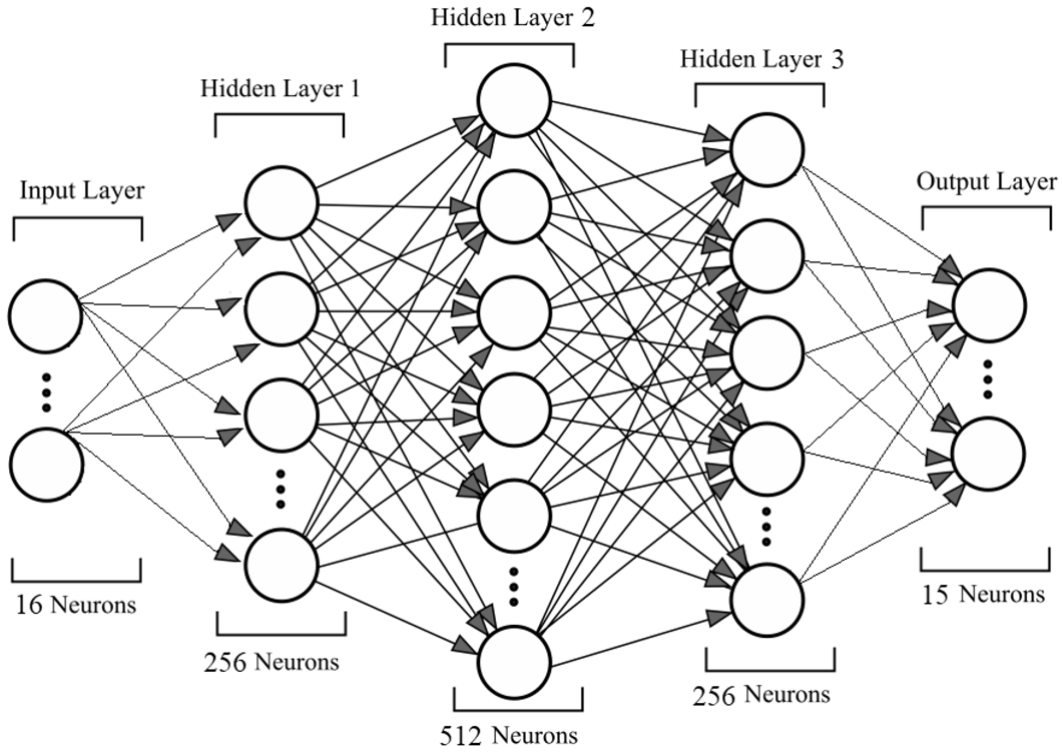
Network structure used for multi-crop land suitability prediction, containing 16 input neurons (1 for each soil-climate-landscape variable), 3 hidden layers, and 9 output neurons (1 for each crop). Output is the estimated volume of crop produced at the pixel level.

A multilayer perceptron (MLP) was used to model the crop yields of barley, canola, flaxseed, lentils, oats, peas, soybeans, corn, and spring wheat across Canada. The output is a continuous multivariate response, and the input are the soil-climate-landscape variables, see Table 1. This is not a spatio-temporal model as it does not consider the variance-covariance structure at the spatial or temporal scales. The perceptron has 16 input neurons, 3 hidden layers with 256, 512, and 256 neurons, respectively, and an output layer with 9 neurons (one for each crop); see Fig. 1. The network also contains a dropout of 95% for each hidden layer and uses the ReLU non-linear function. A small network with a high dropout helped prevent over-fitting as the training set is small. The MLP was trained such that, for a given district during a year, it would predict how much volume of crop is produced in each pixel of spatial resolution in the district where the crop was observed to grow. Using the 16 soil-climate-landscape variables at each pixel as input; a single pixel represents an area of 0.3 km by 0.5 km. The model would then sum up the predictions for all pixels and divide by the total number of pixels in the district, resulting in a yield prediction for crop *c* in district *d* during year *t*, denoted by ${\hat{y}}_{c,d,t}$. In other words,

$$\begin{aligned} {\hat{y}}_{c, d, t} = \frac{1}{n_{c, d, t}} \sum _{i=1}^{n_{c, d, t}} {\hat{y}}_i, \end{aligned}$$

where $c= \{ \text {barley}, \text {canola}, ..., \text {spring wheat} \}$, $n_{c, d, t}$ is the total number of pixels in district *d* for which crop *c* is grown during year *t*, and ${\hat{y}}_i$ is the yield prediction for pixel *i* in district *d* for which crop *c* is grown during year *t* (we omit the subscripts *c*, *d*, *t* here for ease of notation). For example, from Table 2, spring wheat is grown on pixels 1 to 650 which belong to district Alberta 1 for the year 2013. The yield of spring wheat in Alberta 1 for the 2013 harvest is thus predicted by

$$\begin{aligned} {\hat{y}}_{\text {spring wheat}, \text {Alberta 1}, \text {2013}} = \frac{1}{650} \sum _{i=1}^{650} {\hat{y}}_i, \end{aligned}$$

where ${\hat{y}}_i$ is the volume of spring wheat produced at the pixel level of the geospatial images predicted by the MLP. This model is considered semi-supervised learning because the volume of crop produced at the pixel level in a district is not known; the yield is only known at the district level. Training is done by minimizing the error between ${\hat{y}}_{\text {spring wheat}, \text {Alberta 1}, \text {2013}}$ and $y_{\text {spring wheat}, \text {Alberta 1}, \text {2013}}$, where $y_{\text {spring wheat}, \text {Alberta 1}, \text {2013}}$ is the observed yield at the district level.

However, training a model that can simultaneously predict the yield of multiple crops is challenging because only a single crop is grown on a region of farm land during the growing season. In turn, the MLP cannot be trained conventionally since the yield at harvest of all 9 crops must be known for a set of soil-climate-landscape variables at a given location. A solution to this problem is to update the network weights during training according to only the crop whose yield is observed. This is mathematically achieved by zeroing the error between predicted and observed district yields for the 8 unobserved crops by using an indicator function, $I_{\text {observed}}$. Here is an example of the calculation for the loss function when the district yield of oats is known (we omit the subscripts *d*, *t* here for ease of notation):

$$\begin{aligned} \text {Loss} = I_{\text {observed}}^T E = \begin{bmatrix} 0&1&\cdots&0 \end{bmatrix} \begin{bmatrix} | {\hat{y}}_{\text {peas}} - y_{\text {peas}} | \\ | {\hat{y}}_{\text {oats}} - y_{\text {oats}} | \\ \vdots \\ | {\hat{y}}_{\text {corn}} - y_{\text {corn}} | \end{bmatrix} = | {\hat{y}}_{\text {oats}} - y_{\text {oats}} |. \end{aligned}$$

The models were trained using three NVIDIA Pascal P100 GPU in TensorFlow with the Adam optimizer. For all models, L1 loss was minimized during training. The Python programming language was used to develop the model, and QGIS was used to visualize the geospatial data.

### Model evaluation

K-fold cross-validation was used to evaluate the model because the dataset is small. Crop yield data at the SAD level from the period of 2013–2020 was used to generate the entire dataset. However, the SADs from 2013–2016 and 2017–2020 do not match completely. Hence, the SADs selected for the validation set had to overlap between the two time periods. Four SADs were selected and all of the examples belonging to these SADs were used to form the validation set. The examples from the remaining SADs were used to form the training set. Separating training and validation sets by district results in a validation set that is completely unseen to the trained model, and thus, is a better measure of how the model will perform on never before seen regions. Eight models were trained because there are 31 SADs that overlap between the two time periods, and k is set to 4. Even though eight models are trained, fewer than eight models may be evaluated because yield observations may not be available for the districts selected to be in the validation set.

We compare our multi-crop model to single-crop models to confirm the hypothesis that the multi-crop should out perform the single-crop models. The same network architecture was used for the single-crop models. Unfortunately, our multi-crop model cannot be compared to standard machine learning models, such as random forest or LASSO, because our dataset cannot be modelled by conventional supervised learning methods as it is not completely labeled. We get around this by using semi-supervised learning with a non-traditional loss function.

## Results and discussion

### Model evaluation

The dataset is comprised of 2110 observations spanning 9 crops; more details on the dataset are presented in Table 3. The multi-crop model demonstrated an acceptable fit for all crops, except for corn and flaxseed (see Table 4). For corn, the number of unique districts in which it is grown is by far the smallest compared to the other crops (see column No. Unique Districts in Table 3), with corn being almost completely grown in just a small cluster in southern Manitoba, southern Ontario, and southern Quebec. Consequently, it is likely that there was not sufficient soil-climate-landscape variation captured in the dataset for corn to develop an adequately generalizable model, as demonstrated by the poor prediction error. The datasets for barley and oats capture the most variance, followed by canola, spring wheat, peas, flaxseed, and then soy.

For the multi-crop model, K-fold cross validation resulted in a mean error that was about 21% of a crop’s yield range, which is acceptable but certainly not very accurate (see Table 4). This mean error rate is acceptable because the soil-climate-landscape variables alone should not be expected to explain most of the variance in the yield as there are other more difficult to measure variables affecting the yield, such as crop management policies, disease and pests, farming practice, and the type of cultivar grown. Though the model is not highly accurate, it is still useful for understanding how soil-climate-landscape variables affect the suitability of land for agriculture.

Corn and flaxseed were dropped from the analysis because the prediction error was too high for the multi-crop model. It is interesting that the single-crop model for corn performs better than the multi-crop model. This may be due to lucky parameter initialization, or because the single-crop model is focused on learning a set of parameters that work well for corn as opposed to all crops in the dataset. The latter setting raises concerns for over-fitting, especially since the dataset for corn is very limited in number of training examples and in soil-climate-landscape variation (see Table 3). With the exception of corn, the mean absolute error of the single-crop models was often much higher than that of the multi-crop ones. For instance, the mean absolute error of peas was reduced by a factor of 2.82 for the multi-crop model compared to the single-crop model. The errors for oats and soy were about the same for both modelling methods.

Considering a multi-crop model was able to be trained to a degree that outperforms the single crop models suggests that the semi-supervised learning method has great potential for learning multivariate regression tasks when the ground truth values of most classes are not known. In particular, it is useful when the ground truth values cannot be known for all classes for a given set of inputs, such as for yield prediction. Even though only one crop can be grown on a piece of land during a growing season, the multivariate nature of the model allows it to learn a set of weights that work well for predicting all classes. This constraint regularizes the model, thereby improving generalisability.

**Table 3:** Summary of data over the years 2013 to 2020 by crop, consisting of total number of yield observations (examples) at the district level and the total number of unique districts where the crop is grown.

Crop	Total No.	No. Unique	Yield Range	Area (km$^2$)
	Examples	Districts		
Barley	383	66	[22, 90]	120,637
Peas	247	51	[ 0, 63]	97,628
Flaxseed	227	49	[ 0, 33]	22,355
Spring Wheat	242	52	[17, 68]	360,335
Canola	276	52	[19, 47]	328,921
Oats	371	66	[27, 126]	42,608
Soy	224	40	[ 6, 50]	93,502
Corn	102	14	[94, 174]	64,068
**Total**	2110	66	[ 0, 174]	

Yield Range (minimum, maximum) of observed yields for a crop (bushels/acre) is also provided. The Area column denotes the total area of all unique plots of land in Canada that have cultivated the crop between 2013–2020.

**Table 4:** Crop-specific means and standard deviations of mean absolute error of predicted yields (bushels per acre) for the multi-crop and single-crop models respectively computed from K-fold cross-validation.

Crop	Multi-crop model				Single-crop model			
	Mean	SD	No. val sets	Rel. error (%)	Mean	SD	No. val sets	Rel. error (%)
Barley	11.10	2.72	7	16	18.84	4.53	8	28
Peas	8.58	2.30	5	14	24.19	9.09	7	38
Flaxseed	15.66	5.35	6	47	27.92	10.72	6	85
Spring Wheat	9.10	4.64	6	18	20.73	9.65	7	41
Canola	9.33	3.15	6	33	17.71	5.38	7	63
Oats	19.36	3.94	7	20	17.10	2.11	8	17
Soy	11.23	1.94	7	26	13.85	5.48	8	31
Corn	63.57	15.17	7	79	24.01	11.59	8	30

Also provided is the total number of times the validation set contained at least one yield observation for a crop. The relative error is the ratio of the mean of the mean absolute error over the yield range (per Table 3).

### Suitability maps

**Figure 2:**
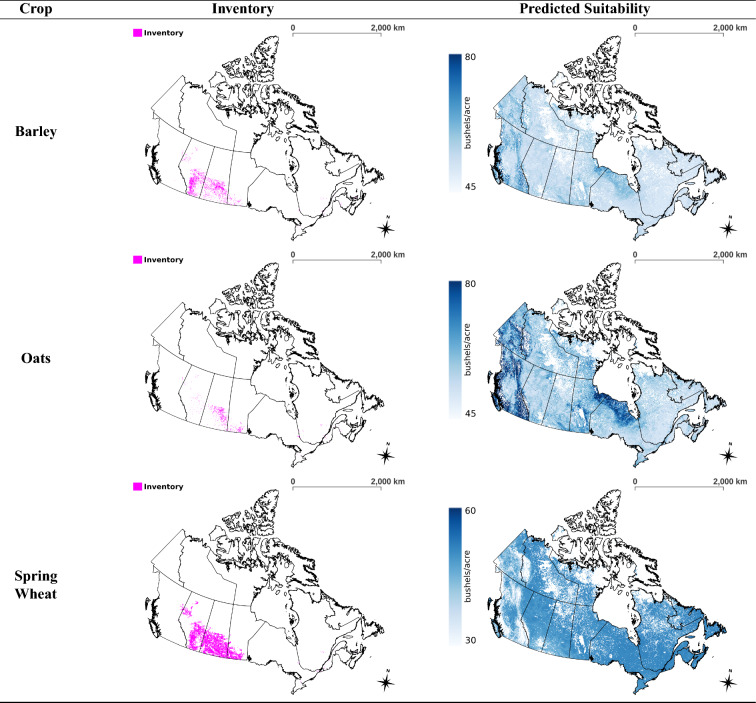

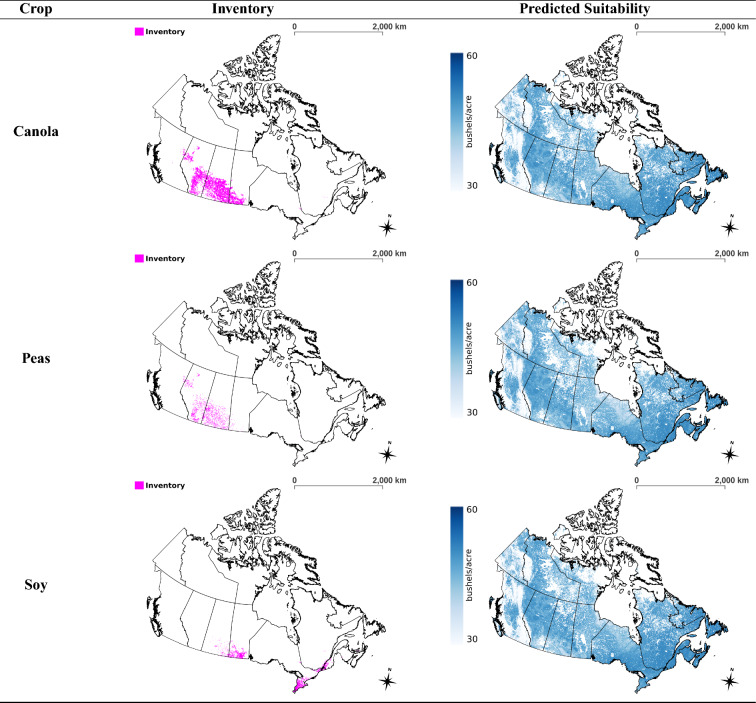
Crop-specific maps of fields from which inventory is obtained (left) and predicted land suitability from multi-crop model (right) across Canada based on years 2013–2020. The suitability colour range for oats and barley represents a difference of 45–80 bushels/acre while that for the remaining maps represents 30 to 60 bushels/acre. This is a multi-page figure. QGIS version 3.30.0 (http://www.qgis.org) was used to create the figure^33^.

The multi-crop model was applied to the soil-climate-landscape variables of Canada for the year 2013 to produce a land suitability map for each of the six crops remaining in the analysis (not including corn and flaxseed). This process was repeated for all years up to 2020, and then the respective set of maps for each crop were averaged to generate a map that represents the current predicted state of land suitability for a crop (see Fig. 2). The enhanced vegetation index was used to mask out regions that are unable to grow sufficient vegetation throughout the year such as the Canadian tundra, see Supplementary Fig. S3 online.

Cultivation in northern Ontario is very limited in regards to crop variety because the crops must be able to withstand dry weather and a short growing season^32^. Barley, oats, and mixed grains are commonly grown in such locations because they are more resistant to variations in soil-climate-landscape conditions^32^. This practice is congruent with our results from Fig. 2 because the maps of barley and oats show that they are capable of being grown across Canada, albeit they are not very suitable to most locations. Spring wheat, though predicted to not be as suitable in western Canada compared to barley and oats, is predicted to be more suitable across Canada compared to the non-grain crops. Furthermore, the crop inventory map of barley provides some evidence to support that barley can be grown in the less steep areas of the Rocky Mountains, see Supplementary Fig. S4 online. These findings suggest that barley and oats are the least sensitive to environmental factors, followed by spring wheat, and then the non-grain crops. In the raw suitability maps of Fig. S3 online, it can be seen that barley and oats are considered to be suitable for agriculture on the steepest slopes of the Rocky Mountains in British Columbia and the Yukon, presumably an extrapolation error. The model learns that barley and oats are not very sensitive to landscape, and it is not aware that vegetation is unable to grow on some of the steepest parts of the Rocky Mountains. Masking does alleviate this error by removing the high slope areas, though for future work, areas not appropriate for growing vegetation can be assigned a yield of 0 to improve model accuracy. Furthermore, the crop inventory map of barley provides some evidence to support that barley can be grown in the less steep areas of the Rocky Mountains, see Supplementary Fig. S4 online.

**Figure 3:**
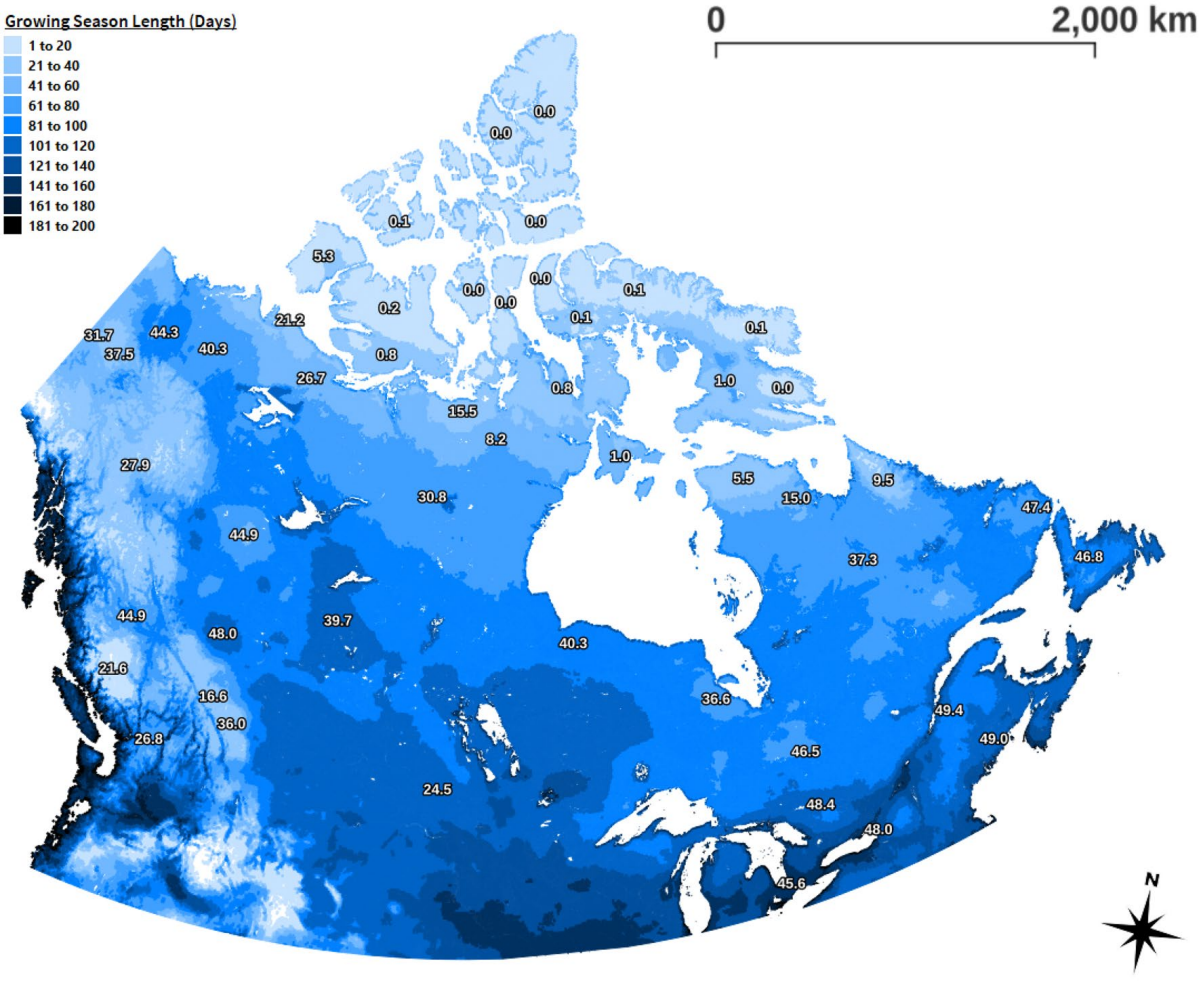
Growing season length (days), averaged over the time period 1981-2010. Numbers in white are predicted yields of peas, averaged over the polygons composing the growing season map and labeled for large polygons. Data credit: Government of Canada (2022). QGIS version 3.30.0 (http://www.qgis.org) was used to create the figure^33^.

The predicted suitability maps of the non-grain crops (see Fig. 2) are observed to correspond well with growing season length, which is in line with the literature^32^. Figure 3 is a map partitioning Canada by growing season length, overlayed with the predicted yield of peas; yield is often low when the growing season length is low. Much of the Yukon and the outer regions of British Columbia are mountainous with short growing seasons and are less suitable for growing peas. The average yield within those regions is low as opposed to zero because vegetation can still be cultivated in some areas. It appears that most of the regions of Canada that have a growing season of at least 61-80 days are suitable for the more environmentally sensitive crops. Of note, the average yield of peas is low in the southern prairies even though the growing season is long due to the presence of a large desert. Ultimately, Fig. 3 serves as a check to help validate the efficacy of the model. Similar trends are present for spring wheat and the remaining non-grain crops as their predicted suitability correlates strongly to that of peas (see Supplementary Fig. S6 online). In contrast, the trend is not as noticeable for barley and oats (see Fig. S5 online), and this is reasonable since they are more capable of being grown in areas with shorter growing seasons^32^. The variability of the predicted crop yields, in the regions for which the respective crops were not harvested during the years 2013–2020, tended to be largest in British Columbia, Yukon and the Northwest Territories (see Supplementary Table S1 online).

The inventory maps in Fig. 2 were created using the annual crop inventory data of Canada by masking out the locations where a select crop was grown during the time period of 2013–2020. A lot of agriculture is done in the southern Prairie provinces, but there is a cluster near the southern Alberta-Saskatchewan border where crops are less cultivated (see Fig. 2). It is interesting that the suitability maps in Fig. 2 predict this area as less suitable for all crops. Perhaps agriculture is avoided in the mentioned area due to the lack of suitability.

**Figure 4:**
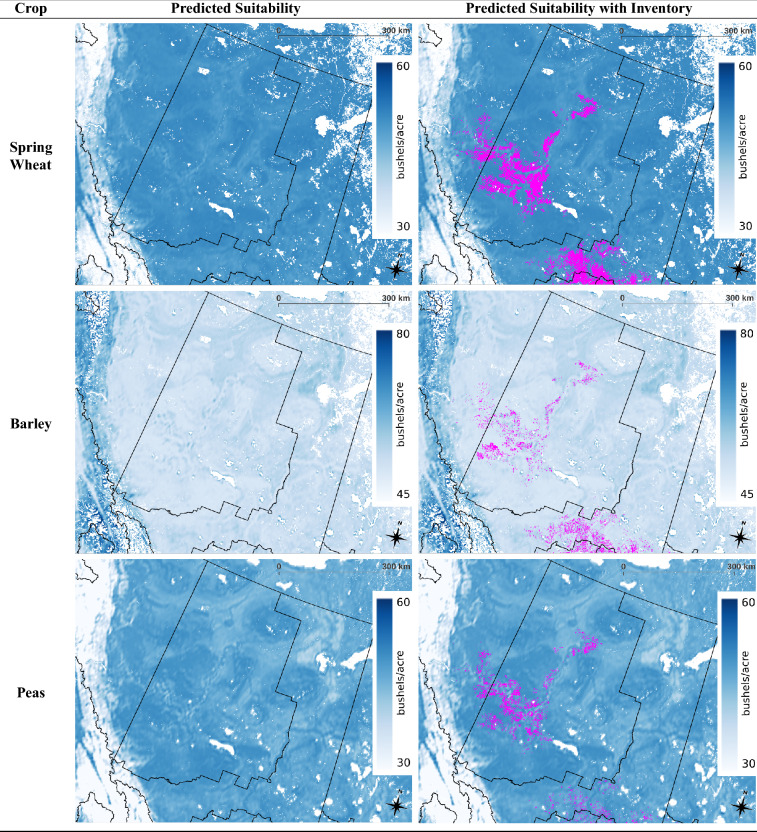
Suitability maps of select crops focused to northern Alberta with SADs Alberta 60 and Alberta 70 boundaries outlined in black. Crops were selected according to if they had sufficient inventory in northern Alberta for visualization purposes. Left: Predicted suitability from multi-crop model (blue). Right: predicted suitability (blue) overlayed with crop inventory (magenta). QGIS version 3.30.0 (http://www.qgis.org) was used to create the figure^33^.

The crop inventory can also be compared to the suitability maps of the SADs that were not used to generate the dataset to provide insights on how the model performs in northern regions that are outside the domain of the training data. The agriculture done in British Columbia, northern Ontario, and northern Quebec is sparse at the large scale level, making it difficult to evaluate these regions. Fortunately, northern Alberta (SADs: Alberta 60 and Alberta 70) is densely packed with agriculture for select crops, see Fig. 4. Figure 4 shows that barley is predicted to be more suitable in the regions where it is cultivated than in the surrounding area, which is an indication that the model can provide sensical predictions in the northern regions of Canada. The same is true for the remaining crops in Fig. 4, though the contrast is not as significant as they are predicted to be quite suitable across northern Alberta.

### Potential limitations

All soil variables, indexed 3–7 on Table 1, are available at 0, 10, 30, 60, 100, and 200 cm depths, but only the values at the 30 cm depth were selected as Fan et al.^34^ showed that several crops have soil depths in the uppermost 30 cm soil profile. However, the median plant root depth ranged from 8.4 to 19.8 cm, so soil levels at 10 cm depth may be preferable. Supplementary Fig. S1 online shows that the values at 10 cm depth and those at 30 cm depth are highly correlated, with soil levels higher at the 10 cm depth. Using the soil values at 10 cm depth instead of the values at 30 cm depth would likely improve our model accuracy somewhat. As such, the results presented here can be thought of as conservative estimates of land suitability. Inclusion of both variables would introduce unnecessary redundancies to the model and would likely have minimal impact on the reported results.

Google Earth Engine’s highest resolution data was used for prediction, with climate data having a spatial resolution of 11+ km$^2$ based on parcel data, while the annual crop inventory data was based on 30 m resolution maps. Despite the vast amounts of data, reaching into the terabytes, it was easily accessible and downloadable onto a laptop for processing and analysis. Higher resolution data could provide more detailed information about the climate patterns at the individual farm level, but would require more storage and computational power. Further, the satellite data, even after downscaling, retained spatial and temporal patterns that were not explicitly exploited during training. Developing a model that exploits the spatio-temporal structure of the data may better capture yield variance caused by the use of different cultivars and farming methodology. Regardless, our model was able to capture much of the spatial variation in its crop land suitability predictions.

There are large regions of Canada that are known to be unsuitable for growing any vegetation and it is common practice to mask out such areas post-analysis^2^. However, these regions could instead be assigned a crop yield of 0 and incorporated into the training to possibly reduce extrapolation error. Crop yield data from other countries could also be added to the dataset enabling a model that has a broader understanding of how different soil-climate-landscape conditions affect the crops. Although the model has demonstrated acceptable extrapolation accuracy for southern Canada, see Rel. Error column in Table 4, and identified trends within the data, such as the correlation between crop yield and growing season length, it can still benefit from additional validation to further improve the model’s credibility for northern Canada. For instance, the model’s reliability in extrapolating can be validated by confirming that the model ranks the importance of each soil-climate-landscape variable similarly to the literature. If the model’s rankings are consistent with established research, this supports that the model has captured the relationships between environmental variables and land suitability.

The gradual increase seen in crop yield over time is partially attributable to advancements in farming techniques and the use of genetically modified crops, rather than solely due to climate change^35^. Such advancements would introduce variability into the data that cannot be explained solely by soil-climate-landscape variables. Certain types of land that may be either highly suitable or unsuitable for farming may not have any recorded yield data due to farmers’ decisions not to cultivate them, possibly because of their remote locations or economic factors. Consequently, the predicted suitability where farmers typically do not grow crops will come with a higher level of uncertainty. However, these disadvantages would be present for any land suitability model, whether data-driven or not because even expert knowledge is inspired by data.

## Conclusions and future work

The appropriateness of land for agriculture in Canada must be evaluated for the current time and the future to facilitate land-use management planning. We developed, from our knowledge, the first data-driven model that simultaneously predicts how suitable land is for growing each of six crops. The model was trained with district level crop yield data, and the output is continuous as opposed to a discrete land suitability class. Our results suggest that the multi-crop model can learn how soil-climate-landscape variables can be predictive of the suitability of land for agriculture, as validated by K-fold cross validation.

The file sizes involved when working with satellite data are so large that reduction of input variables is necessary in order to alleviate computational constraints. Choice of which variables and how they are reduced may introduce some bias into the modelling process but some degree of subjectivity is difficult to avoid. In future work, we aim to make these decisions more data-driven. Variable selection of model inputs for neural networks is an interesting line of research.

Our model predicts that regions in northern Canada are suitable for crop growth, which supports suitability projections under climate change that predict more land in northern Canada will become suitable for agricultural cultivation^2^. These predictions raise questions regarding agricultural land-use change management. A data-driven land suitability model could facilitate a cost-benefit analysis that weighs future food security benefits, economic and trade value, and environmental harm related to cultivating new lands. Our modeling approach has the potential to assist decision-makers in weighing the potential economic and humanitarian benefits against the environmental and social costs of cultivating new lands, including carbon release from clearing and tilling land, harm to biodiversity hotspots, impacts to indigenous territory, and detrimental effects to water resources.

## Supplementary Information

Supplementary Information.

## Data Availability

The datasets used and/or analysed during the current study available from the corresponding author on reasonable request.
