# Supplementary material for: Simultaneous multi-crop land suitability prediction from remote sensing data using semi-supervised learning
Source: Sci Rep. 2023 Apr 26;13:6823. doi: 10.1038/s41598-023-33840-6 (PMC10133274; doi:10.1038/s41598-023-33840-6)
Supplement: Supplementary file 1 — Supplementary Information. [file 41598_2023_33840_MOESM1_ESM.pdf]

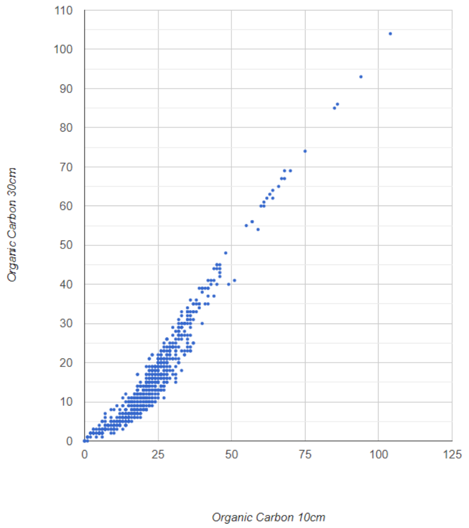

(a) Organic Carbon

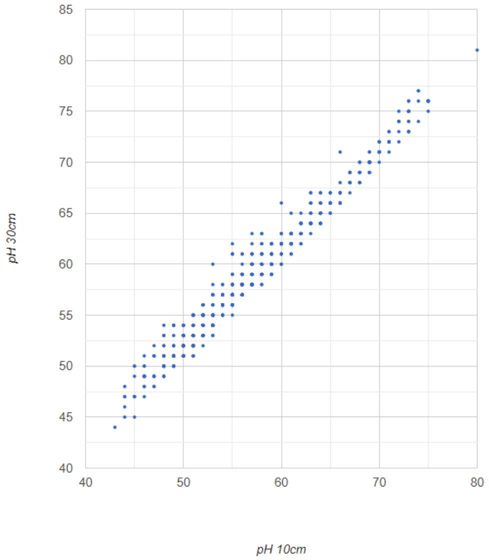

(b) pH

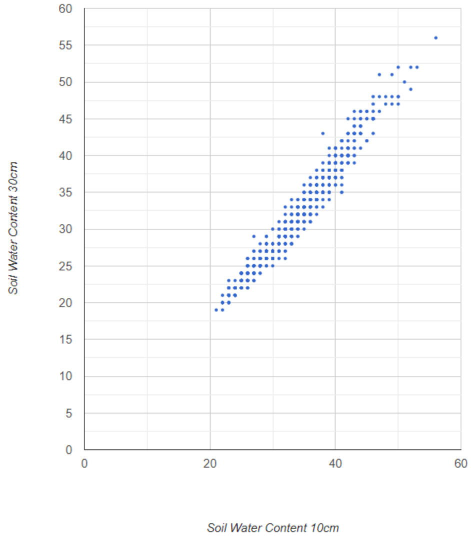

(c) Soil Water Content

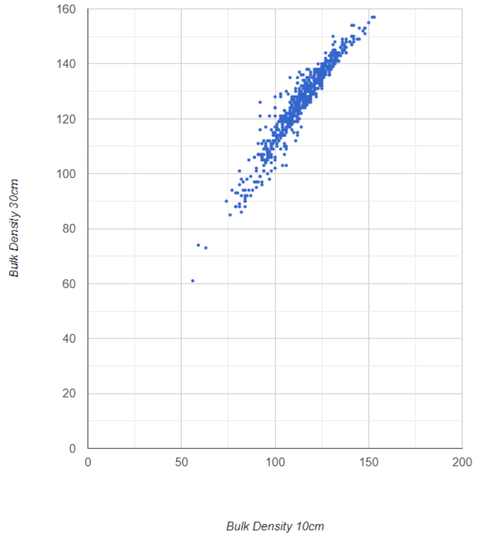

(d) Bulk Density

Figure S1. A thousand random points were sampled from the study area and the organic carbon, pH, water content, and bulk density level of the soil at a depth of 10cm was plotted against the level of the soil at a depth of 30cm. The units of organic carbon, pH, water content, and bulk density are g/kg, logarithmic units x 10, %, and kg/m^3, respectively.

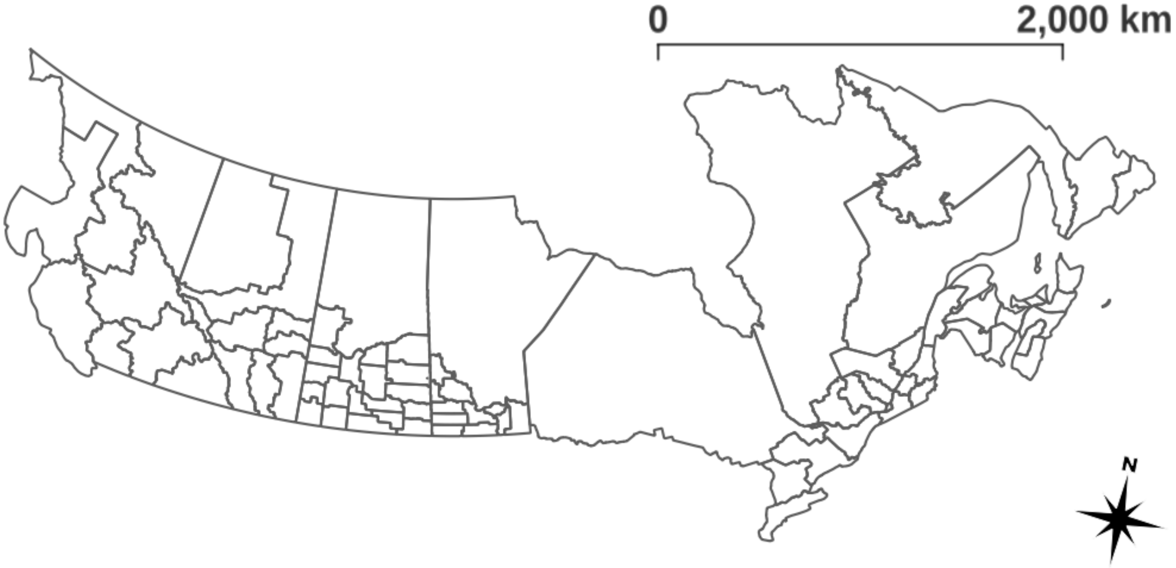

Figure S2. The Small Area Data regions of Canada for the years 2013 to 2016. QGIS version 3.30.0 (http://www.qgis.org) was used to create the figure[32].

Table S1. The mean ± standard deviation of the predicted yield (bushels per acre), by province, after removing regions where the crop was actually grown.

Province	Barley	Peas	Spring Wheat	Canola	Oats	Soy
Newfoundland and Labrador	52.5±2.0	48.5±2.2	50.7±1.2	48.6±2.9	55.8±4.8	49.1±2.5
Prince Edward Island	54.5±3.3	46.7±2.7	50.7±0.5	46.3±3.5	60.4±7.5	48.1±2.3
Nova Scotia	52.6±2.3	48.2±2.1	50.6±0.5	48.1±2.5	56.1±5.3	49.3±1.7
New Brunswick	52.0±1.9	48.9±1.8	50.5±0.5	48.9±2.1	54.2±4.3	49.6±1.4
Quebec	52.8±1.9	48.1±1.8	50.7±0.7	48.6±1.8	55.9±4.1	48.8±1.7
Ontario	55.4±3.5	45.0±3.7	50.6±0.6	46.3±2.9	61.8±8.2	46.7±3.0
Manitoba	56.9±3.2	43.6±3.4	50.1±0.9	44.9±2.9	64.7±7.4	44.8±3.0
Saskatchewan	54.6±2.3	46.0±2.2	48.8±1.9	46.4±2.8	58.3±4.2	46.0±3.2
Alberta	54.1±2.5	46.2±3.4	48.6±2.5	45.8±4.8	57.7±5.5	45.9±4.4
British Columbia	59.2±6.7	37.3±10.5	42.5±7.3	33.4±13.7	71.9±16.5	34.4±13.2
Yukon	57.6±3.9	40.2±6.4	44.4±4.6	36.9±8.8	67.2±9.5	37.8±8.3
Northwest Territories	55.6±3.1	44.9±4.5	49.3±3.5	44.9±6.3	61.7±7.1	45.0±6.0
Nunavut	57.0±2.4	45.0±1.9	50.6±0.9	45.6±2.1	64.4±5.0	44.9 ±2.4

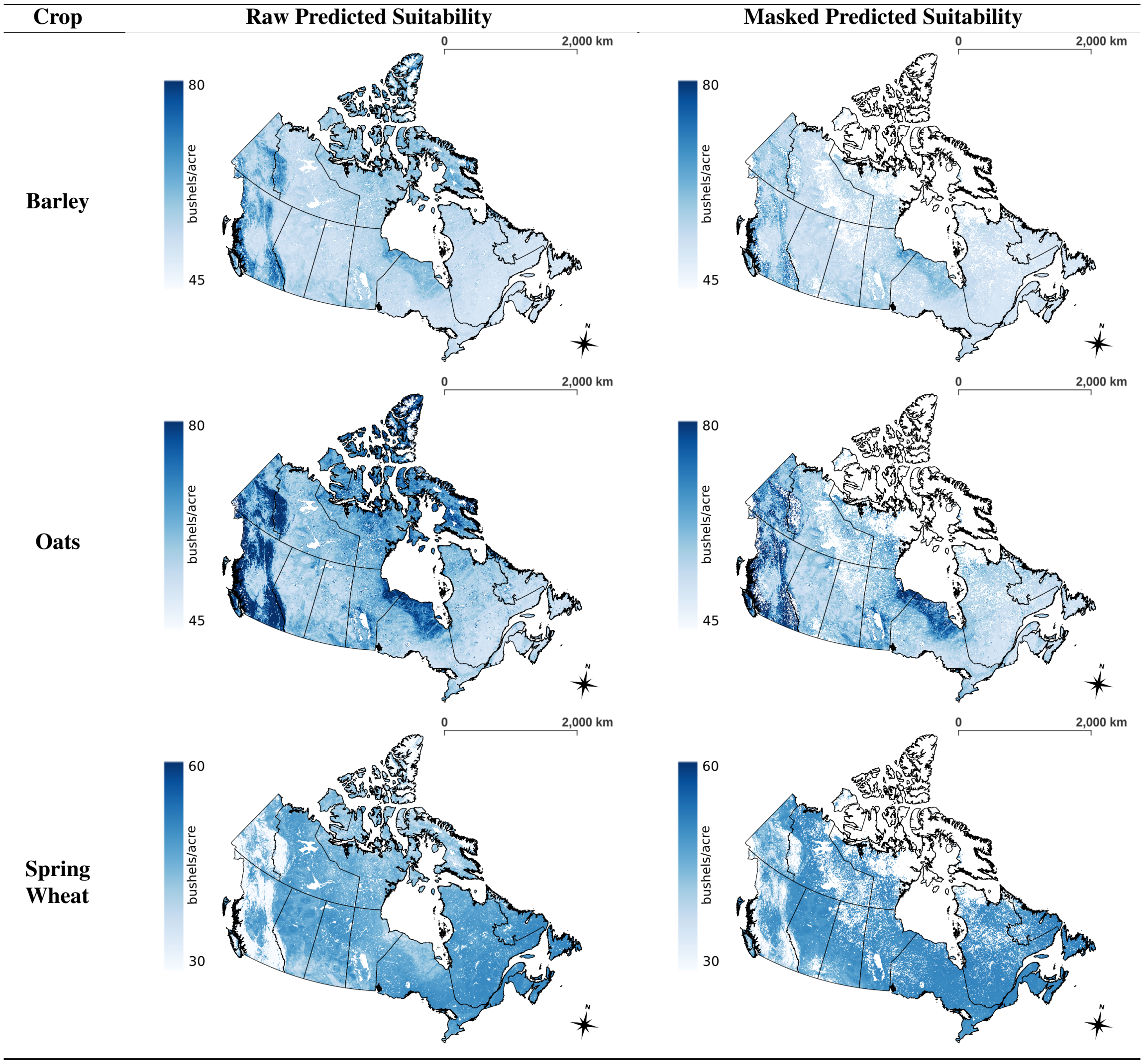

Figure S3. Crop-specific maps of multi-crop model predicted land suitability across Canada, both unmasked (left) and masked (right) using vegetation data. The suitability colour range for oats and barley represents a difference of 45 to 80 bushels/acre while that for the remaining maps represents 30 to 60 bushels/acre. This is a multi-page figure. QGIS version 3.30.0 (http://www.qgis.org) was used to create the figure[32].

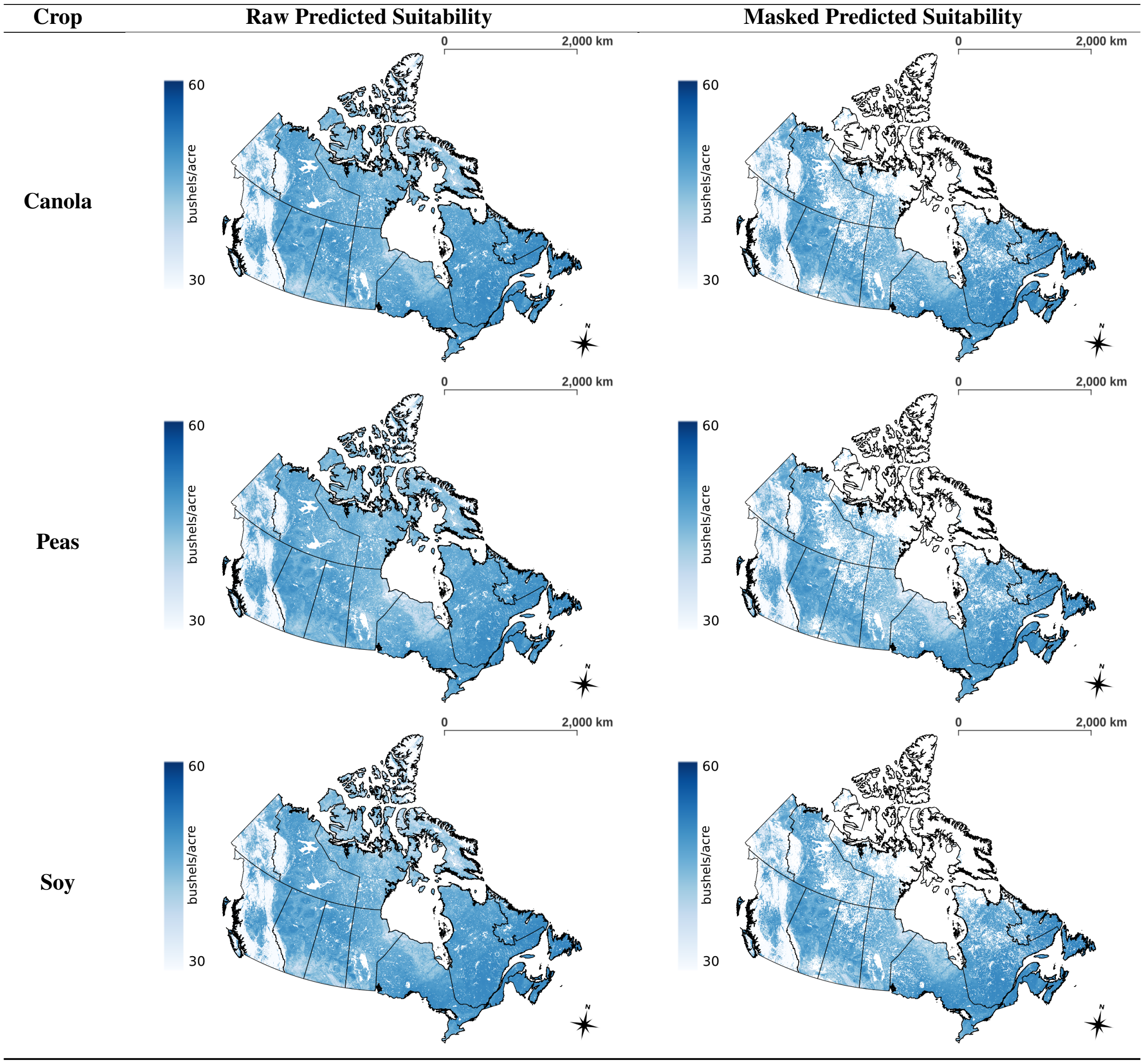

Figure S3. Crop-specific maps of multi-crop model predicted land suitability across Canada, both unmasked (left) and masked (right) using vegetation data. The suitability colour range for oats and barley represents a difference of 45 to 80 bushels/acre while that for the remaining maps represents 30 to 60 bushels/acre. This is a multi-page figure. QGIS version 3.30.0 (http://www.qgis.org) was used to create the figure[32].

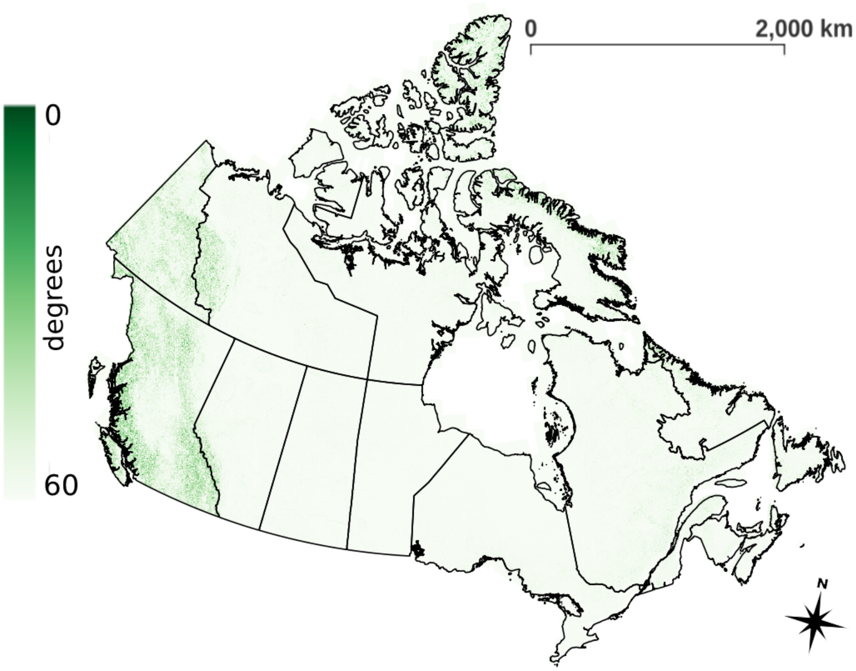

(a) Slope map

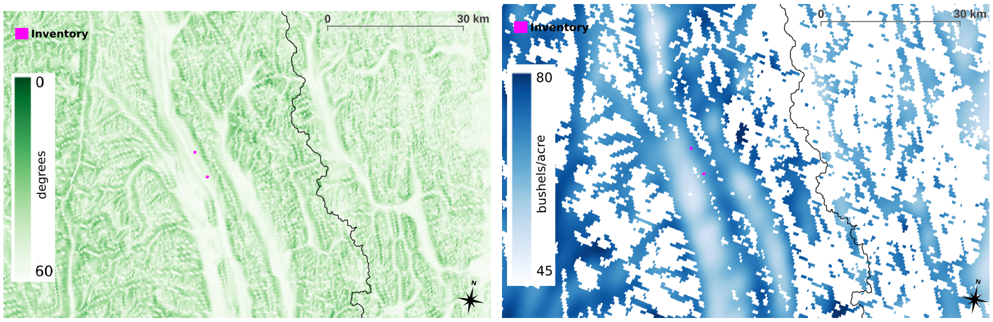

(b) Barley inventory with slope map **(c)** Barley inventory with barley suitability map

Figure S4. (a) Map of slope across Canada. Focusing on the Rocky Mountains along the southern British Columbia and Alberta border: (b) slope, and (c) predicted suitability overlayed with locations of two fields growing barley as shown in magenta. The colour ranges are 0 to 60 degrees for the slope maps and 45 to 80 bushels/acre for the suitability map. QGIS version 3.30.0 (http://www.qgis.org) was used to create the figure[32].

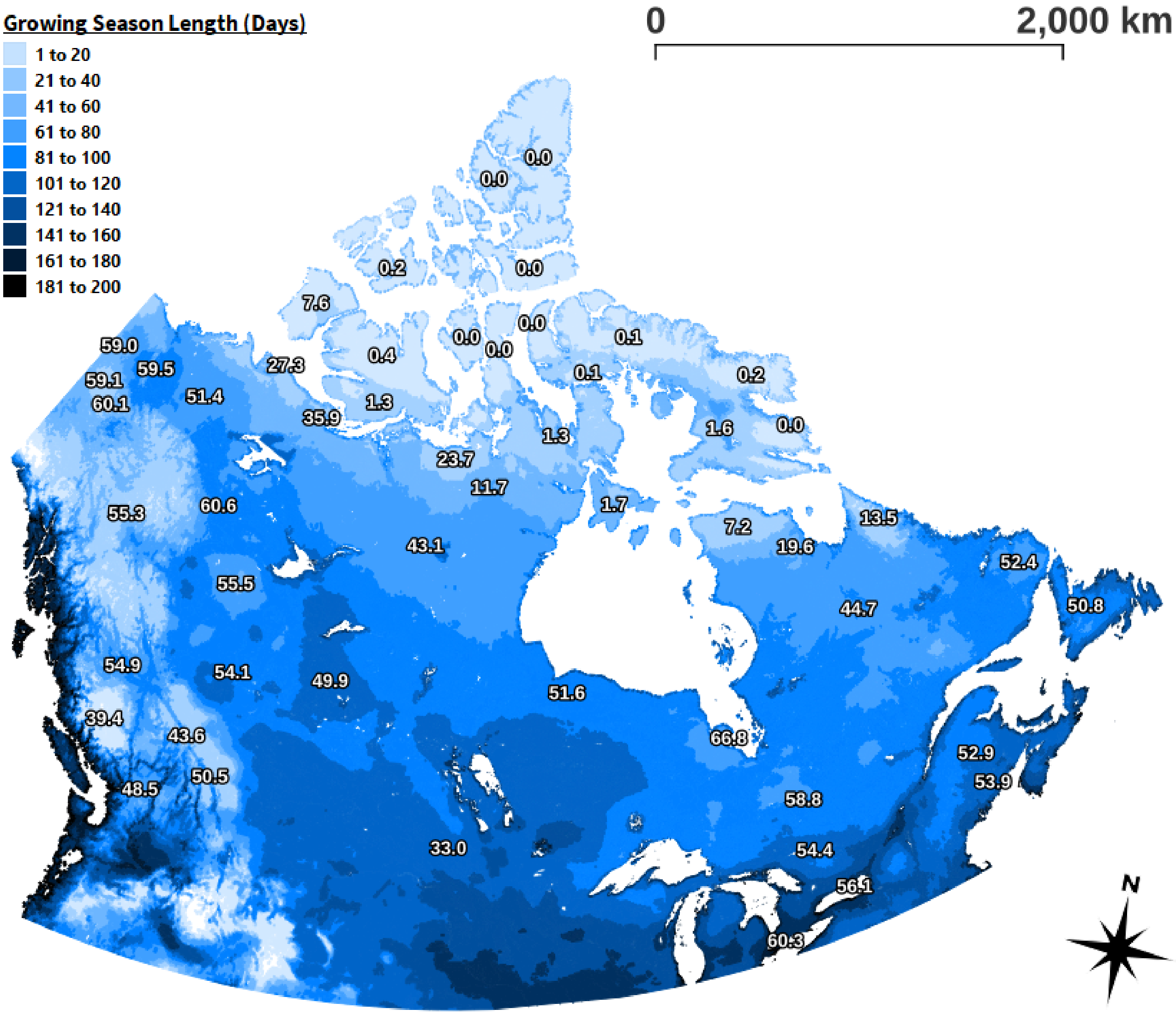

Figure S5. Growing season length (days), averaged over the time period 1981–2010. Numbers in white are predicted yields of oats, averaged over the polygons composing the growing season map and labeled for large polygons. Data credit: Government of Canada (2022). QGIS version 3.30.0 (http://www.qgis.org) was used to create the figure[32].

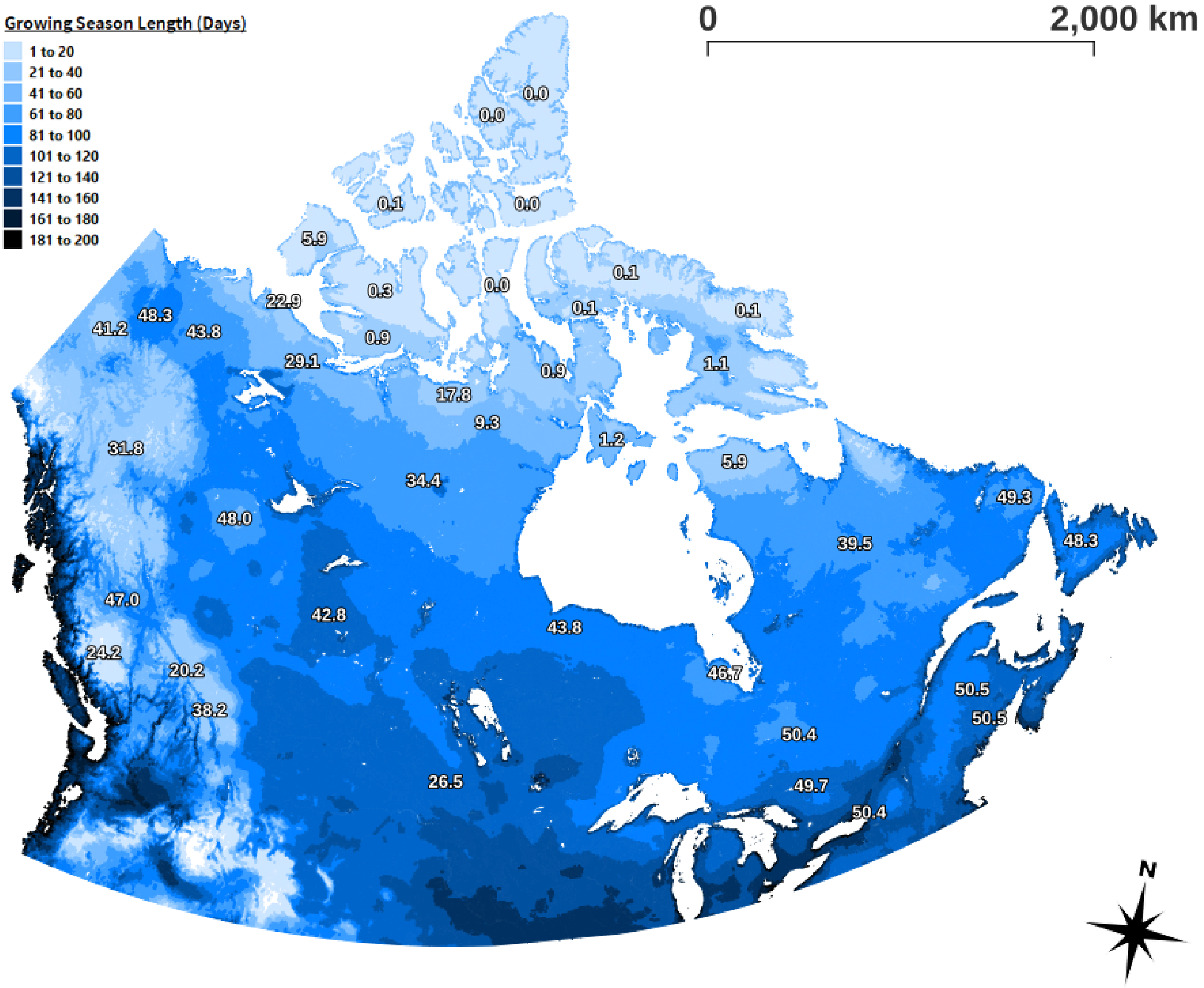

Figure S6. Growing season length (days), averaged over the time period 1981–2010. Numbers in white are predicted yields of spring wheat, averaged over the polygons composing the growing season map and labeled for large polygons. Data credit: Government of Canada (2022). QGIS version 3.30.0 (http://www.qgis.org) was used to create the figure[32].
